# Supplementary material for: Immunomodulation of Selective Naive T Cell Functions by p110δ Inactivation Improves the Outcome of Mismatched Cell Transplantation
Source: Cell Rep. 2015 Feb 5;10(5):702–10. doi: 10.1016/j.celrep.2015.01.002 (PMC4542309; doi:10.1016/j.celrep.2015.01.002)
Supplement: Document S1. Figures S1 and S2 [file mmc1.pdf]

Cell Reports
Supplemental Information

Immunomodulation of Selective Naive T Cell Functions by p110δ Inactivation Improves the Outcome of Mismatched Cell Transplantation

Jean-Marc Doisne, Christian M. Hüber, Klaus Okkenhaug, and Francesco Colucci

Figure S1 – Related to Figure 3

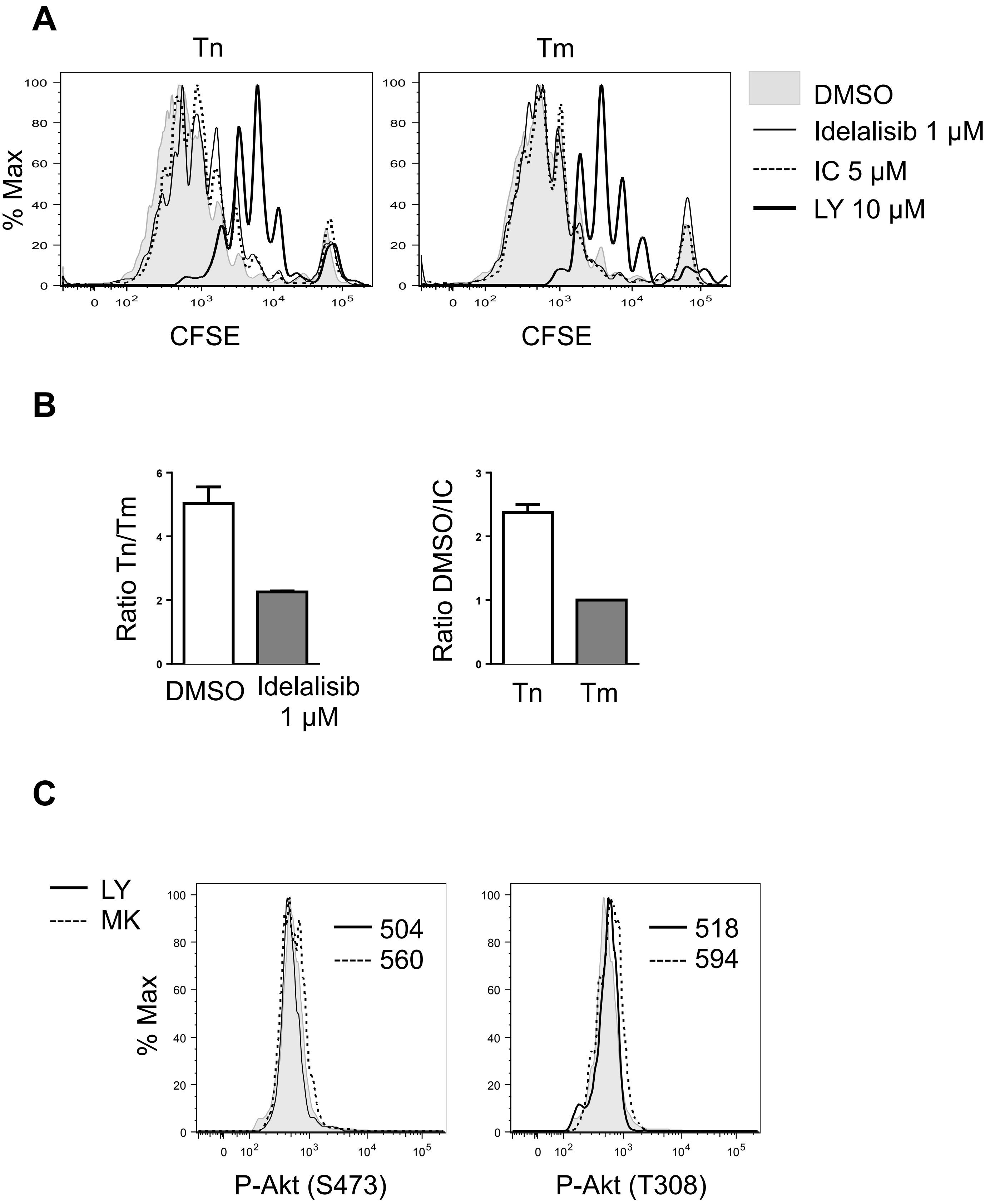

Figure S2 – Related to Figure 4

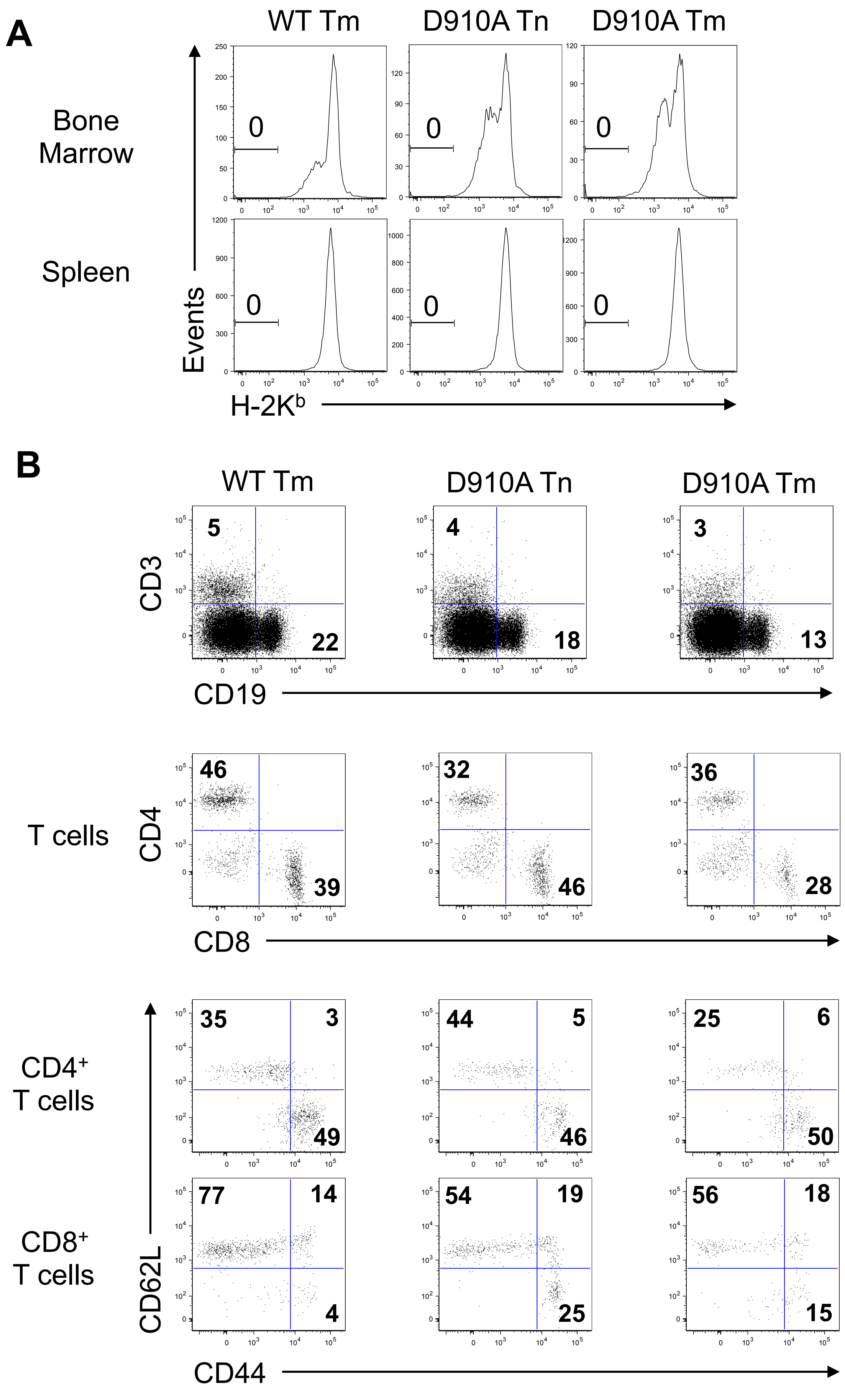

SUPPLEMENTAL LEGENDS

Figure S1 – Related to Figure 3. (A) Same experiment as in **Fig 3A** including treatments with DMSO (vehicle), Idelalisib (1 µM), IC87114 (5 µM) and LY294002 (10 µM). Results are representative of two independent experiments. **(B)** Alloreactive T cells were generated as in **Fig 3C** in the presence of DMSO or Idelalisib (1 µM). Results are presented as in **Fig 3C** and are representative of two independent experiments. **(C)** Alloreactive Tn generated in culture in the presence of DMSO were pre-treated with LY294002 (10 µM) or MK-2206 (5 µM) for 30 min then stained for P-Akt (Ser473) or P-Akt (Thr308). Results are representative of two independent experiments.

Figure S2 – Related to Figure 4. (A-B) Surviving mice from WT Tm group, D910A Tn group and D910A Tm group (depicted in Fig. 4) were analyzed by flow cytometry at day 91. **(A)** Histograms show representative examples of the absence of A20 cells (H-2K^{b-} CD19^{+}) in the BM and the spleen. **(B)** Dot plots show representative examples of leukocyte composition in the BM: T and B cells (upper panel), CD4^{+}/CD8^{+} T cells (middle panel) and their differentiation phenotype (bottom panel).
