# Supplementary material for: Immunomodulation of Selective Naive T Cell Functions by p110δ Inactivation Improves the Outcome of Mismatched Cell Transplantation
Source: Cell Rep. 2015 Feb 5;10(5):702–10. doi: 10.1016/j.celrep.2015.01.002 (PMC4542309; doi:10.1016/j.celrep.2015.01.002)
Supplement: Document S2. Article plus Supplemental Information [file mmc2.pdf]

Cell Reports

Article

Immunomodulation of Selective Naive T Cell Functions by p110δ Inactivation Improves the Outcome of Mismatched Cell Transplantation

Graphical Abstract

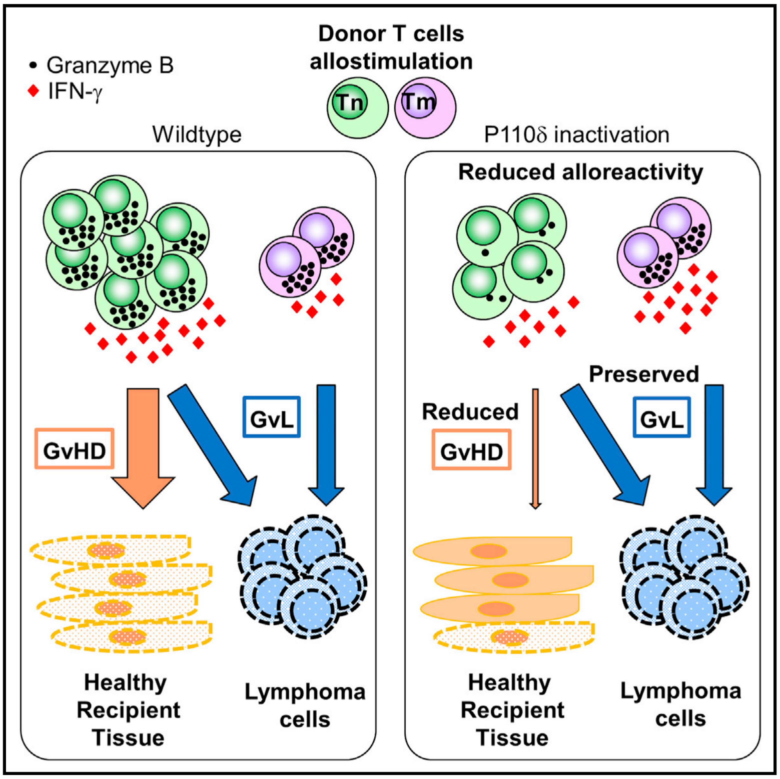

Highlights

- Genetic p110δ inactivation in donor naive T cells mitigates GvHD in mice
- Pharmacological p110δ inactivation in human T cells reduces alloreactivity

Authors

Jean-Marc Doisne, Christian M. Hüber, Klaus Okkenhaug, Francesco Colucci

Correspondence

jmd83@medschl.cam.ac.uk

In Brief

Using a mouse model of fully mismatched hematopoietic cell transplantation, Doisne et al. show that p110δ inactivation interferes with selective naive T cell functions and favorably sways the balance between graft versus leukemia and graft versus host disease. Furthermore, inactivation of p110δ in human lymphocytes reduces T cell allorecognition.

Doisne et al., 2015, Cell Reports *10*, 702–710
February 10, 2015 ©2015 The Authors
http://dx.doi.org/10.1016/j.celrep.2015.01.002

Immunomodulation of Selective Naive T Cell Functions by p110δ Inactivation Improves the Outcome of Mismatched Cell Transplantation

Jean-Marc Doisne,[1,*] Christian M. Hüber,[1] Klaus Okkenhaug,[2] and Francesco Colucci[1]
[1]Department of Obstetrics and Gynaecology, University of Cambridge School of Clinical Medicine, NIHR Cambridge Biomedical Research Centre, Cambridge CB2 0SW, UK
[2]Laboratory for Lymphocyte Signaling and Development, Babraham Research Campus, The Babraham Institute, Cambridge CB22 3AT, UK
*Correspondence: jmd83@medschl.cam.ac.uk
http://dx.doi.org/10.1016/j.celrep.2015.01.002
This is an open access article under the CC BY license (http://creativecommons.org/licenses/by/3.0/).

SUMMARY

Allogeneic hematopoietic stem cell transplantation (HSCT) can treat certain hematologic malignancies due to the graft versus leukemia (GvL) effect but is complicated by graft versus host disease (GvHD). Expression of the p110δ catalytic subunit of the phosphoinositide 3-kinase pathway is restricted to leukocytes, where it regulates proliferation, migration, and cytokine production. Here, in a mouse model of fully mismatched hematopoietic cell transplantation (HCT), we show that genetic inactivation of p110δ in T cells leads to milder GvHD, whereas GvL is preserved. Inactivation of p110δ in human lymphocytes reduced T cell allorecognition. We demonstrate that both allostimulation and granzyme B expression were dependent on p110δ in naive T cells, which are the main mediators of GvHD, whereas memory T cells were unaffected. Strikingly, p110δ is not mandatory for either naive or memory T cells to mediate GvL. Therefore, immunomodulation of selective naive T cell functions by p110δ inactivation improves the outcome of allogeneic HSCT.

INTRODUCTION

Allogeneic hematopoietic stem cell transplantation (HSCT) is a challenging procedure used to treat certain malignancies. The challenge is to minimize the complications and maximize the benefits of the genetic disparity between donors and recipients. Mismatched T cells in the graft provide alloreactivity against cancer cells (graft versus leukemia [GvL]); however, mismatched T cells also react against host tissue antigens, leading to graft versus host disease (GvHD). The devastating effects of GvHD are limited by immunosuppressive treatment of patients, but current regimens increase the risk of relapse and opportunistic infections. Combination therapies that harness the power of immune cells and the potential of new drugs to manipulate selective lymphocyte functions (Houot et al., 2011; McDaniel et al., 2012) may be considered to improve the outcome of allogeneic HSCT (Li and Sykes, 2012), and recent work suggests that interfering with proximal T cell signaling may be an effective strategy (Valenzuela et al., 2009; Haarberg et al., 2013). In mice, naive T cells mediate both GvL and GvHD, whereas memory T cells mediate only GvL (Dutt et al., 2011; Ruggeri et al., 2002); thus, small-molecule inhibitors that target selective functions in naive T cells may improve the outcome of allogeneic HSCT.

Phosphoinositide 3-kinase (PI3K) enzymes are crucial components of lymphocyte development and function (Okkenhaug, 2013). The catalytic subunits p110γ and p110δ are predominantly expressed in hematopoietic cells (HCs). It has been shown that p110δ is important for development, differentiation, and regulation of T cell subsets (Patton et al., 2007; Okkenhaug, 2013). Emerging evidence suggests that p110δ is an attractive pharmacological target to modulate both unwanted immune responses and certain blood cancers (Soond et al., 2010; Billottet et al., 2006; Sujobert et al., 2005). Indeed, p110δ-selective inhibitors are currently being tested in clinical trials to treat autoimmunity, allergy, and lymphoid malignancies. For example, idelalisib (GS-1101, CAL-101), which is derived from IC87114, is being tested for treating non-Hodgkin's lymphoma, Hodgkin's lymphoma, and chronic lymphoid leukemia (Furman et al., 2014).

Our results show that p110δ inactivation interferes with selective naive T cell functions and favorably sways the balance between GvL and GvHD during the course of allogeneic HSCT.

RESULTS

Alleviated GvHD

Mouse models of acute GvHD show different levels of severity, depending on the number and timing of allogeneic cell injection and whether total splenocytes or only T cell subsets are injected. We set up a mouse model of fully mismatched HSCT (B6 into BALB/c mice), in which purified T cells are injected soon after lethal irradiation and cause acute severe GvHD and death of recipient mice within 7 days.

To assess the impact of p110δ inactivation on T cells in GvHD, we used transgenic (*p110δ*^{D910A}) knockin mice, which carry a loss-of-function point mutation in the kinase domain of p110δ (Okkenhaug et al., 2002). We lethally irradiated BALB/c mice, in which we then transferred myeloprotective cells from T cell depleted (TCD) B6 bone marrow cells (BMCs) alone (BM group)

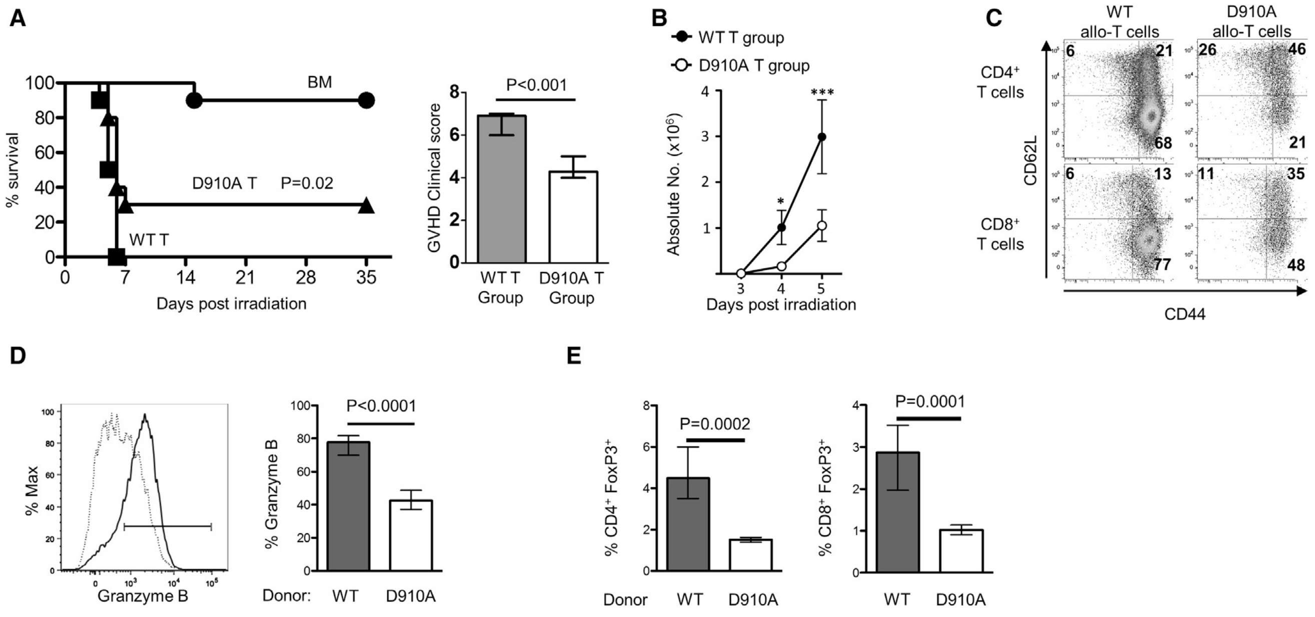

Figure 1. Inactivation of p110δ Reduces GvHD Severity and Impairs Allogeneic T Cell Functions In Vivo

(A) Survival curve of two combined independent experiments where lethally irradiated BALB/c hosts were intravenously (i.v.) injected with either 5 × 10^6 TCD-BMCs only (BM group, n = 10) or 5 × 10^6 TCD-BMCs and 1 × 10^6 control T cells (WT T group, n = 10) or 1 × 10^6 D910A T cells (D910A T group, n = 10). The difference in survival between the two latter groups is significant (p = 0.02; Mantel-Cox test). The graph on the right panel shows the combined results of two independent experiments quantifying GvHD clinical score of BALB/c mice receiving $p110\delta^{WT/D910A}$ (control) T cells (gray column; n = 10) or D910A T cells (white column; n = 7) evaluated at the endpoint (loss of 20% bodyweight) (mean ± range, unpaired t test).

(B) Spleens from lethally irradiated BALB/c mice injected with allogeneic T cells from WT (WT T group) or D910A (D910A T group) mice were harvested at day 3, 4, and 5. Absolute numbers of donor T cells were calculated on the basis of flow cytometry analysis (n = 3–5; mean ± range; unpaired t test; *p = 0.029, ***p = 0.001).

(C) Representative dot plots of $CD4^+$ and $CD8^+$ T cell differentiation profile based on CD62L and CD44 expression 4 days after hematopoietic cell transplantation (HCT).

(D) At day 5, allogeneic $CD8^+$ T cells from WT (solid line) or D910A (dotted line) mice were analyzed for GzmB expression by flow cytometry. Data are from one experiment with n = 5 mice in each group (mean ± range, unpaired t test).

(E) The percentage of $CD4^+$ (left) and $CD8^+$ (right) Tregs were assessed at day 5 post-HCT based on the expression of FoxP3 measured by intracellular flow cytometry (n = 5; mean ± range; unpaired t test).

or along with allogeneic T cells from B6 wild-type (WT) (WT T group) or B6 $p110\delta^{D910A}$ (D910A T group) mice (Figure 1A). Most (nine out of ten) mice in the BM group recovered fully from irradiation and survived until the endpoint (35 days). All mice in the WT T group had to be culled within 6 days after having rapidly developed clinical signs of severe GvHD reaching the 20% weight loss endpoint and a clinical score of 7 on a scale of 8 (Figure 1A). In the D910A T group, seven out of ten mice developed a milder form of GvHD (clinical score 4) but had to be culled because the weight loss had reached 20%. Remarkably, three out of ten mice in this group did recover and survived for more than 35 days (Figure 1A). Thus, inactivation of p110δ alleviates clinical signs and improves survival in a mouse model of acute and severe GvHD.

Analysis of host spleens within 5 days posttransplantation revealed 3- to 6-fold fewer donor T cells in mice from the D910A T group (0.16 ± 0.06 × 10^6 at day 4 and 1.05 ± 0.34 × 10^6 at day 5) as compared to the WT T group (1.01 ± 0.37 × 10^6 at day 4 and 2.99 ± 0.80 × 10^6 at day 5) (Figure 1B). Furthermore, donor T cells in the D910A T group contained more $CD62L^+CD44^{hi}$ central memory T cells and fewer $CD62L^-CD44^{hi}$ effector memory T cells compared to the WT T group (Figure 1C). Cleavage of CD62L upon allorecognition was inhibited by p110δ inactivation, as expected from previous work (Sinclair et al., 2008). Granzyme B (GzmB) is a key effector molecule in allogeneic $CD8^+$ T cells. Fewer $GzmB^+$ cells were found within $p110\delta^{D910A}$ allogeneic $CD8^+$ T cells (43% ± 5% versus 78% ± 5%), and the cells expressing GzmB did so at lower intensity (Figure 1D).

Regulatory T cells (Tregs) modulate GvHD and GvL (Hoffmann et al., 2002; Edinger et al., 2003). PI3K signaling plays a role in Treg generation and function (Soond et al., 2012). We reasoned that p110δ-inactivated T cells could contain more Tregs, therefore explaining the reduced GvHD. We found instead that mice receiving T cells from B6 $p110\delta^{D910A}$ mice had ~3-fold fewer $CD4^+$ and $CD8^+$ Tregs (1.5% ± 0.1% versus 4.8% ± 1% and 1% ± 0.09% versus 2.9% ± 0.6%, respectively; Figure 1E), the latter being generated specifically during allogeneic HSCT in mice (Robb et al., 2012). These results suggest that the beneficial effect of p110δ inactivation on GvHD is unlikely to be mediated by Tregs. We therefore reasoned that p110δ inactivation may rather inhibit expansion, differentiation, and acquisition of effector functions in allogeneic effector T cells.

Preserved GvL

The main reason to transplant mismatched T cells is that they mediate killing of tumor cells, and we therefore assessed the

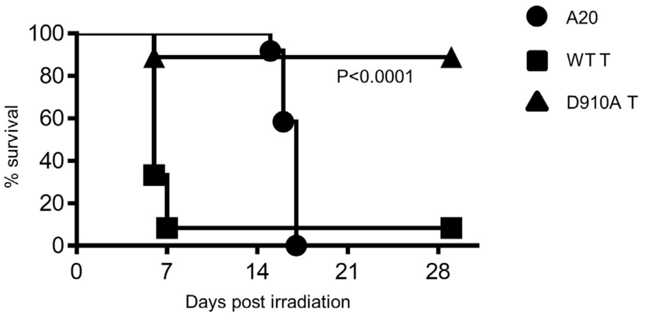

Figure 2. Inactivation of p110δ Preserves GvL

Lethally irradiated BALB/c hosts were i.v. injected with 5 × 10^6 TCD-BMCs and 5 × 10^4 A20 (lymphoma) cells (A20 group, n = 12), 5 × 10^6 TCD-BMCs plus 5 × 10^4 A20 cells in the presence of B6 T cells from either WT (WT T group, n = 12) or D910A (D910A T group, n = 9) mice. The endpoint for this experiment was 25% weight loss, rather than 20% as in Figure 1. The p value (Mantel-Cox test) indicates statistical difference between the WT T group and the D910A T group. Data are from two experiments with n = 4–8 mice in each group.

impact of p110δ-inactivation on GvL. We lethally irradiated three groups of BALB/c mice, in which we then cotransferred myeloprotective cells (as in the previous experiment) and syngeneic A20 lymphoma cells alone (A20 group) or along with allogeneic T cells from B6 WT (WT T group) or B6 $p110\delta^{D910A}$ (D910A T group) mice (Figure 2). Instead of culling recipient mice at 20% of bodyweight loss (Figure 1), we decided to set the endpoint at 25% weight loss. This was because we noticed that mice receiving p110δ-inactivated T cells showed a lower GvHD clinical score and that the surviving mice regained weight quickly and survived in apparent good health. This suggests that the beneficial effect of p110δ inactivation in allogeneic T cells may be delayed.

All mice in the A20 group succumbed to lymphoma within 17 days, and 11 out of 12 mice in the WT T group developed acute GvHD caused by WT T cells and succumbed within just 1 week. In striking contrast, eight out of nine mice in the D910A T group recovered after an initial weight loss and survived both mild GvHD caused by p110δ-inactivated T cells (thus confirming and expanding the results of the previous experiment) and tumor growth up until day 30 posttransplant, when they were culled for analysis (Figure 2). No or very few residual lymphoma cells were found in the spleen, liver, and bone marrow of the eight surviving mice in the D910A group (data not shown), showing that selective p110δ inactivation not only alleviates GvHD but also preserves robust GvL. On aggregate, these results show that p110δ inactivation in donor lymphocytes separates harmful GvHD from beneficial GvL.

Reduced Alloreactivity of Naive T Cells In Vitro

Both naive and memory T cells mediate GvL, but GvHD is mediated by naive T cells only (Dutt et al., 2011). We hypothesized that the mechanisms underlying the effects of p110δ inactivation on GvHD and GvL could be related to differential requirements for p110δ signaling in naive and memory T cells. To test this hypothesis, we analyzed the effect of selective pharmacological inhibition of p110δ by IC87114 (IC) or idelalisib on naive ($CD62L^+CD44^{lo}$) and memory ($CD44^{hi}$) WT T cells in vitro. Pharmacological inactivation of p110δ reduced proliferation of naive $CD8^+$ T cells, which underwent one round of division less than when incubated with the vehicle (seven rather than eight divisions). In contrast, memory $CD8^+$ T cell proliferation was not inhibited by pharmacological inactivation of p110δ (Figures 3A and S1A). The pan-PI3K inhibitor LY294002 (LY) strongly reduced proliferation of both subsets (Figures 3A and S1A). Alloreactive naive $CD8^+$ T cells displayed a similar $CD62L^+CD44^{hi}$ profile that was also observed in vivo in transgenic cells (Figure 1C), supporting the notion that p110δ inactivation—genetically or pharmacologically—interferes with differentiation of alloreactive naive T cells (Figure 3B).

In order to assess the significance of these results in a tractable cellular model relevant to HSCT, we measured the effects of pharmacological p110δ inactivation in T cell responses to tumor-primed allogeneic bone marrow-derived dendritic cells (BMDCs). Naive T cells were more responsive to allogeneic cells than memory T cells, but p110δ inactivation halved the number of alloreactive naive T cells and compromised their differentiation, whereas memory T cells were unaffected by p110δ inactivation using IC or idelalisib (Figures 3C and S1B). GzmB expression was strongly suppressed by p110δ inactivation in naïve, but not memory, T cells (Figure 3D). Consistent with reduced PI3K signaling in naive T cells, but not memory T cells, we found that p110δ inactivation reduced the phosphorylation of Akt at both sites S473 and T308 in naïve, but not memory, T cells (Figures 3E and S1C). When primed T cells were restimulated with A20 cells or A20-pulsed BMDCs, memory T cells produced more interferon-γ (IFN-γ) than naive T cells. Moreover, upon p110δ inactivation during restimulation, only in memory T cells was IFN-γ production enhanced (Figure 3F). Thus, alloreactive naive and memory T cells have different p110δ signaling requirements.

Reduced Alloreactivity of Naive T Cells In Vivo

To confirm our hypothesis, allogeneic naive and memory T cells were sorted and used as effector cells in our mouse model of severe GvHD. We lethally irradiated five groups of BALB/c mice, in which we then cotransferred myeloprotective cells and syngeneic A20 lymphoma cells alone (A20 group) or along with either allogeneic naive (WT Tn group) or memory (WT Tm group) T cells from B6 WT or along with either allogeneic naive (D910A Tn group) or memory (D910A Tm group) T cells from B6 $p110\delta^{D910A}$ (Figure 4). In unseparated T cells, naive T cells are the vast majority (85%). We injected 1 × 10^6 purified naive T cells. Rather than injecting 0.15 × 10^6 cells, which is the number of memory T cells one should inject to recapitulate the Tn/Tm ratio of unseparated T cells, we opted for injecting an excess of memory T cells (0.5 × 10^6 cells; that is, more than three times greater than the Tm equivalent of unseparated cells), in order to exclude any effect due to disparity of cell numbers.

The majority of the mice (12/14) in the WT Tn group had to be culled within 7 days because of GvHD. Among the two remaining mice, one was culled due to signs of severe late GvHD (weight loss, skin rush). All mice in the D910A Tn group developed milder GvHD and survived longer, demonstrating that allogeneic naive T cells are less potent inducers of severe GvHD when p110δ is inactivated. Some other mice (5/12) developed clinical signs of late GvHD (skin rush, diarrhea) and had to be culled in accordance with the UK Home Office regulation. None of the mice in the

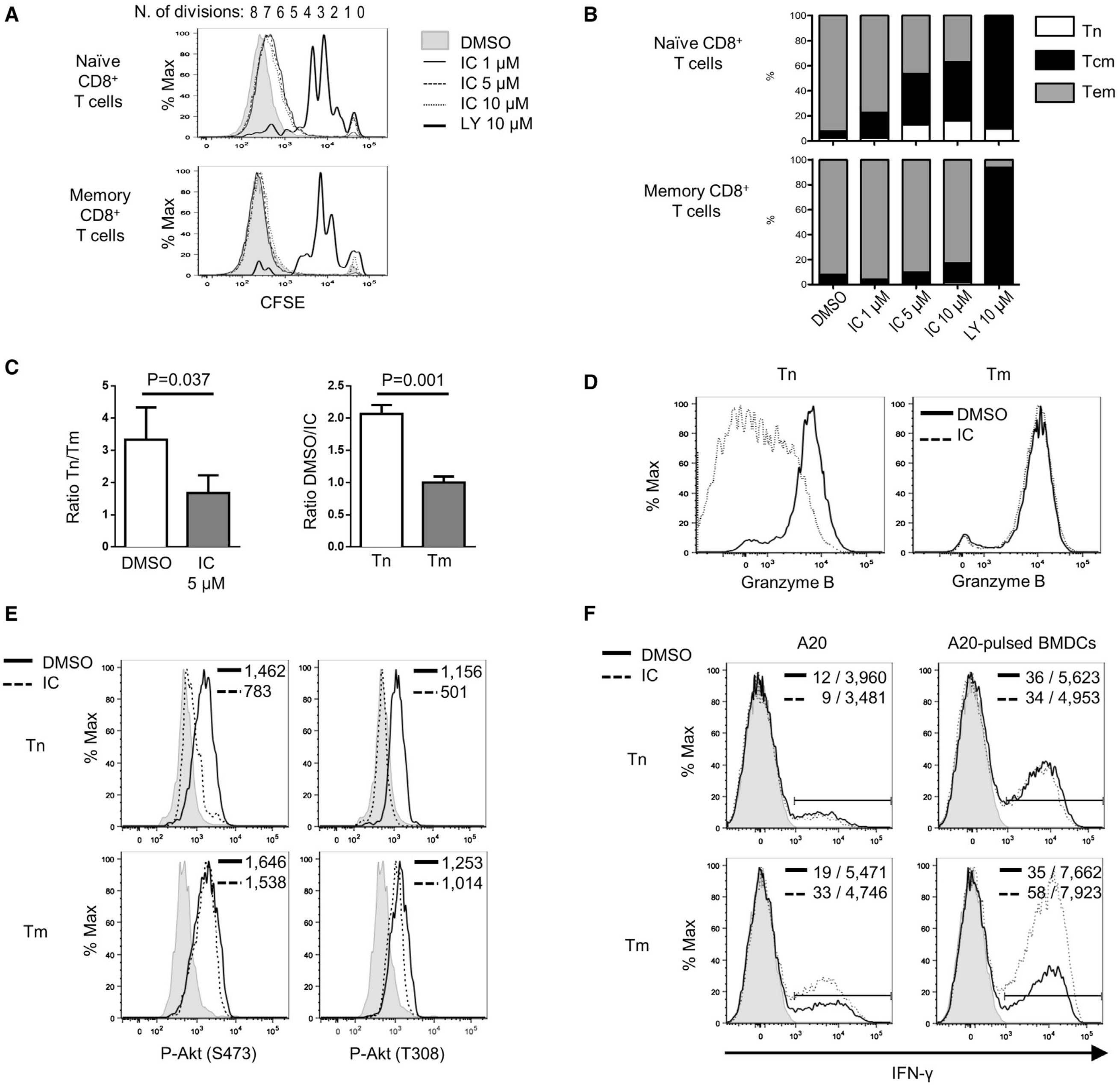

Figure 3. Inactivation of p110δ Inhibits Allogeneic Naive T Cell Proliferation and Differentiation In Vitro

(A and B) B6 sorted naive and memory T cells were labeled with CFSE and cocultured with LPS-matured and irradiated BALB/c BMDCs at a 1:1 ratio with DMSO vehicle control or the indicated doses of IC87114 (IC) or LY294002 (LY). At day 5, proliferation (A) and differentiation (B) of $CD8^+$ T cells were quantified. Differentiation phenotype is based on CD62L and CD44 expression as follows: naive (Tn; $CD62L^+CD44^{lo}$), central memory-like (Tcm; $CD62L^+CD44^{hi}$), and effector memory-like (Tem; $CD62L^-CD44^{hi}$). Results are representative of three independent experiments.

(C–F) T cells come from the cultured alloreactive T cells generated in vitro in presence of DMSO or IC. These cells were stimulated first with irradiated BALB/c BMDCs loaded with A20 lysate for 5 days, then expanded with anti-CD3 and anti-CD28 + rmIL-2/rmIL-7 for 2 days and maintained in a low dose of rmIL-2 and rmIL-7 in the presence of DMSO or IC during the whole process. The level of GzmB (D) and P-Akt (E) were assessed at the end of this process. (C) After 5 days of priming, Tn and memory (Tm, $CD44^{hi}$) T cell absolute numbers were evaluated. Tn/Tm ratios were calculated either in DMSO or in IC (5 μM) (left), and DMSO/IC ratios were determined for each subset (right). Results are from four independent experiments (mean ± range, paired t test). (D) GzmB expression was assessed in Tn and in Tm primed and expanded in the presence of DMSO (solid line) or IC (dotted line). Results are representative of three independent experiments. (E) Tn and Tm cultured in the presence of DMSO (solid line) or IC (dotted line) were stained for P-Akt (Ser473) or P-Akt (Thr308) and analyzed by flow cytometry. Results are representative of two independent experiments. (F) Tn (top) or Tm (bottom) cultured in the presence of DMSO (solid line) or IC (dotted line) were stimulated for 5 hr with A20 cells (left) or A20-pulsed BALB/c BMDCs (right). Intracellular IFN-γ was stained and analyzed by flow cytometry. The percentages and geometric mean fluorescence intensities (GMFIs) of IFN-γ^+ cells are depicted on each histogram. Data are representative of two independent experiments.

See also Figure S1.

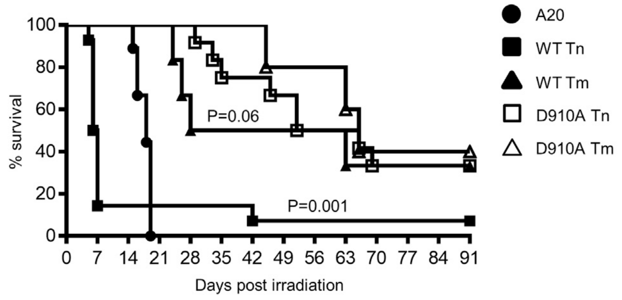

Allo-T cell injected	GvHD		Tumor		GvHD- and Tumor-free at 91d
	≤7d	>7d	<21d	>21d	
None (n=9)	0	0	100%	0	0
WT Tn (14)	86%	7%	0	0	7%
D910A Tn (12)	0	42%	0	25%	33%
WT Tm (6)	0	0	0	67%	33%
D910A Tm (5)	0	0	0	60%	40%

Figure 4. Inactivation of p110δ Inhibits Allogeneic Naive T Cells In Vivo

Lethally irradiated BALB/c hosts were i.v. injected with 5×10^6 TCD-BMCs and 5×10^4 A20 (lymphoma) cells (A20 group, n = 9), 5×10^6 TCD-BMCs plus 5×10^4 A20 cells in the presence of B6 naive T cells (1×10^6 cells) from either WT (WT Tn group, n = 14) or D910A (D910A Tn group, n = 12) mice, or in the presence of B6 memory T cells (0.5×10^6 cells) from either WT (WT Tm group, n = 6) or D910A (D910A Tm group, n = 5). The table summarizes the cause of death for each group: acute GvHD (≤7 days), delayed GvHD (>7 days), tumor (<21 days), or delayed tumor (>21 days). The last column shows the overall survival rate at day 91. The endpoint for this experiment was 25% weight loss. The p values (Mantel-Cox test) compare the WT Tn group with the D910A Tn group and the WT Tm group with the D910A Tm group. Data are from two experiments with n = 5–14 mice in each group. See also Figure S2.

WT Tm group (six out of six) or the D910A Tm group (five out of five) showed any clinical signs of acute or late GvHD. These results confirm that allogeneic memory T cells are unable to induce GvHD, even if the number of memory T cells injected was 3-fold greater than the equivalent of unseparated Tm/Tn cells.

All mice in the A20 group (nine out of nine, no allogeneic T cell grafted) died of lymphoma within 19 days. The GvL effect was observed in the WT Tn group (two remaining mice) and in most of the mice (9/12) of the D910A Tn group, demonstrating that naive T cells lacking functional p110δ are able to mediate GvL. Memory T cells are known to mediate GvL (Dutt et al., 2011). Effectively, a GvL effect could be observed in mice that had received either WT or D910A Tm cells. We noticed a trend for delayed lymphoma development in the D910A Tm group, suggesting a stronger GvL effect (Figure 4). Thus, GvL mediated by p110δ-inactive memory T cells may be more robust. Allogeneic memory T cells upon restimulation produced more IFN-γ when p110δ was inactivated (Figure 3F). This is in line with a report that IFN-γ promotes GvL (Yang et al., 2011) and may explain the more efficient GvL response mediated by p110δ inactivation in memory T cells.

Finally, analysis of the surviving mice after 91 days revealed complete clearance of A20 lymphoma cells in the BM and in the spleen, confirming successful GvL response (Figure S2A). Reconstitution and persistence of engrafted donor HCs occurred in all groups in the BM and in the spleen (Figure S2B and not shown).

These results demonstrate that naive T cells require p110δ in vivo to cause GvHD, but not to mediate GvL. Moreover, memory T cells do not cause GvHD and mediate a more potent GvL when p110δ is inactivated. Tumors are cleared in surviving mice, and the hematopoietic compartment appears to develop normally.

Reduced Alloreactivity in Human T Cells

To validate the results obtained in murine cells, we assessed the impact of p110δ inactivation in human T cell allorecognition in an adapted mixed lymphocyte reaction (MLR) assay using HCs from the blood of healthy donors in the presence of either LY or IC (Figure 5A). The pan-PI3K inhibitor LY reduced the T cell alloresponse by 68%–99% in four out of four donors, whereas the p110δ-selective inhibitor IC reduced it to a lesser extent (47%–68%) in three out of four donors. T cells of one donor (donor D) exhibited low basal alloresponses to two independent stimulator HCs. LY, but not IC, further reduced these responses. We then sorted naive ($CD3^+CD25^-CD45RA^+CD45RO^-CCR7^+$) and memory ($CD3^+CD25^-CCR7^-$ and $CD3^+CD25^-CD45RA^-CD45RO^+CCR7^+$) human T cells to test whether p110δ inactivation had a differential impact on alloresponses in the two subsets (Figure 5B). The results showed that naive T cells and memory T cells proliferated similarly after allostimulation and that alloreactivity of both subsets was inhibited upon p110δ inactivation with IC. This result contrasts with what we found in mouse T cell subsets. However, similarly to what we found in mouse T cell subsets, human naive T cells displayed a more marked inhibition of GzmB expression upon p110δ inactivation than memory T cells (Figure 5C). The results suggest that, despite the interindividual variations, p110δ does modulate allorecognition of HCs in human T cells. The impact of p110δ inactivation on human naive and memory T cell subsets is different in comparison to mice in that both human subsets require functional p110δ to proliferate in response to allostimulation. However, the impact on GzmB expression is comparable in both species in that naive T cells are more affected in both human and mouse T cells.

DISCUSSION

The PI3K/mammalian target of rapamycin pathway is a promising pharmacological target to treat leukemia (Janes et al., 2010). P110δ is an attractive target because it is expressed nearly exclusively by hematopoietic cells (both immune cells and malignant cells), where it regulates selective functions (Ghigo et al., 2010). We show here that p110δ inactivation reduces proliferation and delays differentiation of naïve, but not memory, allogeneic T cells, leading to low GzmB expression. Inhibition of allogeneic naive T cells reduces GvHD severity and

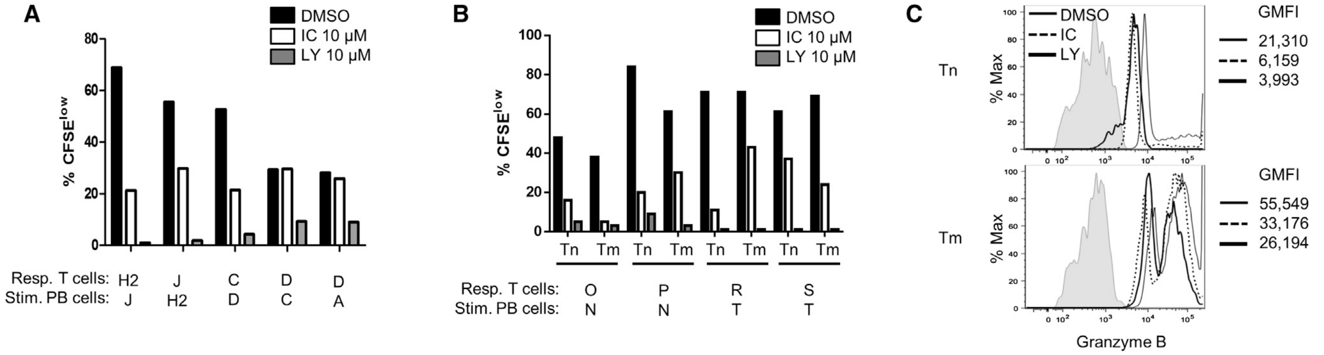

Figure 5. Inactivation of p110δ Inhibits Allogeneic Human T Cell Activation

(A) Healthy donor PBMCs were labeled with CFSE and cocultured with stimulator cells, i.e., irradiated PBMCs from another healthy donor. Mixed lymphocyte cultures at 1:1 ratio were set up in the presence of DMSO vehicle control, IC (10 μM), or LY (10 μM). After 6 days, proliferation of allogeneic T cells was quantified by enumerating the % of $CFSE^{low}$ cells. The histogram graph represents five combinations of responding and stimulator cells from five individual healthy donors whose identity is marked by a capital letter.

(B) A similar experiment was performed using sorted naive and memory T cells from four different donors as responder cells.

(C) Granzyme B expression (GMFI) was measured by flow cytometry in $CD8^+$ allogeneic T cells at day 6 of the MLR assay. Histograms are representative of two independent experiments.

improves the outcome of allogeneic HSCT. More importantly, p110δ inactivation preserves GvL.

The nature of the antigens, the costimulatory signals, and the type of antigen-presenting cells (APCs) involved in GvHD remain to be defined in order to determine how p110δ inactivation contributes to decrease GvHD severity. Nevertheless, modulation of proximal T cell receptor (TCR) signaling appears to be key to alleviate GvHD and preserve GvL. Indeed, PKCα and PKCθ inactivation in T cells also dissociates GvHD from GvL and preserves antimicrobial immunity (Valenzuela et al., 2009; Haarberg et al., 2013). Mouse studies reveal that memory T cells are only able to mediate GvL and not GvHD, whereas naive T cells mediate both (Dutt et al., 2011). Memory T cells that sustain GvHD can be generated in vivo during GvHD, but these are postmitotic $CD8^+$ T cells derived from naive donor T cells (Zhang et al., 2005b, 2005a). We cannot exclude that these cells contribute to GvHD in our model. Indeed, we show that allogeneic naive T cells give rise to memory/effector T cells during GvHD, whereas allogeneic memory T cells are unable to induce GvHD. Unlike memory T cells, naive T cells need costimulation following the engagement of the TCR/CD3 complex, and indeed blocking CD28 and ICOS reduces GvHD (Taylor et al., 2005; Li et al., 2011, 2013). Importantly, CD28 and ICOS are dispensable for GvL (Ohata et al., 2002; Hubbard et al., 2005). P110δ signals downstream of CD28, which, in turn, enhances TCR-mediated p110δ activation (Garçon et al., 2008). P110δ is also downstream of the ICOS signaling pathway, although a PI3K-independent pathway has also been described (Li et al., 2013). We show also that phosphorylation of Akt S473 and Akt T308 is inhibited in naive T cells, whereas memory T cells exhibit normal levels of phosphorylation. Blocking p110δ is emerging as an effective strategy to inhibit costimulatory signals on allogeneic naive T cells in order to reduce GvHD. Akt activity following alloactivation of memory T cells seems to be independent of p110δ signaling. Further studies are needed to decipher the nature of the receptors and the exact signaling pathways involved in alloreactivity of memory T cells.

P110δ inactivation inhibits allostimulation of naive T cells, resulting in low levels of GzmB expression at day 5 postinjection. This may contribute to reduce GvHD. Indeed, other studies have shown a role for both GzmB and perforin in $CD8^+$ T cells during GvHD in fully major histocompatibility complex (MHC)-mismatched transplantation (Graubert et al., 1997). *GzmB*$^{-/-}$ mice can mediate stronger GvL with high production of IFN-γ (Bian et al., 2013). IFN-γ production is known to promote GvL (Yang et al., 2011), and we show that allogeneic memory T cells generated in vitro produce more IFN-γ than naive T cells, and IFN-γ production is further enhanced when p110δ is inactivated. It will be also interesting to study the impact of enhanced IFN-γ production on natural killer (NK) cells and macrophages. Like NK and NKT cells, memory T cells store preformed GzmB mRNA, allowing for quicker responses upon activation (Stetson et al., 2003). Further studies will define if the inhibition is at the level of gene expression or mRNA translation. In line with our results, a recent report shows that T cells from *p110δ*$^{-/-}$ mice express low GzmB when activated with anti-CD3ε but, in contrast to our data, antitumor T cell responses were impaired (Putz et al., 2012). One possible explanation for the discrepancy may be routed in both the genetic makeup and the nature of the tumor tissue, allogeneic lymphoma in our case and syngeneic carcinoma in the other. Altogether, these results show that p110δ inactivation does not impair memory T cell functions during allogeneic responses and is fully compatible with GvL.

In addition, p110δ also regulates T cell migration and is a key factor for CD62L cleavage; therefore, its inactivation interferes with T cell recirculation (Sinclair et al., 2008). Abortive alloresponses and different patterns of migration of memory T cells have been proposed to explain their inability to induce GvHD (Chen et al., 2007). Despite the very rapid onset of acute GvHD precluding the possibility to assess T cell infiltration in target organs in our model, our results confirm that p110δ inactivation interferes with T cell activation, proliferation, and differentiation upon stimulation by allogeneic APCs and that p110δ inactivation

does not interfere with GvL responses. Donor T cells are also important for the engraftment of donor HCs. Given that p110δ inactivation can impair T cell trafficking, engraftment and reconstitution may be less efficient. However, our data suggest this is not the case in our model, given that engraftment and reconstitution were not impaired in surviving mice analyzed after 3 months.

P110δ inactivation in human T cells does not result in the dichotomy between alloresponses in naive and memory T cells observed in mice, as both subsets are affected in humans while only naive T cells are affected in mice. Although alloreactivity was inhibited in both human subsets, the decrease in GzmB expression was greater in naive T cells, comparably to mouse T cells. Memory T cells from human healthy donors are not comparable with memory T cells from mice kept in pathogen-free conditions, and this might explain, at least in part, the different impact of p110δ inactivation on alloresponses of human and mouse memory T cells. Moreover, the role of naive and memory T cells in humans during GvHD and GvL is also less clear than in mice, where a clear dichotomy exists and memory T cells do not cause GVHD. Nevertheless, in vitro evidence suggests that most of the leukemia-reactive and GvHD-inducer human T cells come from the naive compartment. Memory T cells exhibit lower alloreactivity and poor antileukemia response but may transfer memory responses against pathogens to the recipient (Distler et al., 2011). Indeed, recent studies focus on depleting $CD45RA^+$ T cells, which include naive and late differentiated T cells, to reduce GvHD and keep a memory response (Touzot et al., 2014; Chan et al., 2014; Teschner et al., 2014). Additional work on human T cell subsets will aim to assess the impact of p110δ inactivation on antileukemia response and memory responses to pathogens.

Our study is a proof of principle that inhibition of p110δ can favorably sway the balance between GvHD and GvL in a model of a strong acute GvHD and a highly aggressive tumor during fully MHC-mismatched hematopoietic cell transplantation. In the clinic, most allogeneic HSCTs are performed in the haploidentical settings, so future studies will evaluate the impact of p110δ inactivation in haploidentical mouse models to mimic the situation in human patients.

We propose that p110δ inactivation reduces GvHD severity by decreasing allogeneic responses of naive T cells and by interfering with allogeneic T cell recirculation. GvL neither depends on p110δ in naive nor in memory T cells. Indeed, p110δ inactivation does not interfere with effector functions and allostimulation of memory T cells.

Imatinib (Gleevec) has revolutionized the treatment of chronic myeloid leukemia. BRAF inhibitors and anti-CTLA-4 and anti-PD-1 monoclonal antibodies are changing the landscape of cancer treatments (Eagle and Trowsdale, 2007; Chapman et al., 2011; Mellman et al., 2011; Wolchok et al., 2013; Hamid et al., 2013). Our results suggest that p110δ inhibitors could be added to the pharmacological arsenal to improve the outcome of allogeneic HSCT for the treatment of leukemia.

EXPERIMENTAL PROCEDURES

Mice

C57BL/6 (here referred to as B6) and BALB/c mice were purchased from Charles River Laboratories. $P110\delta^{D910A}$ (C57BL/6) strain was previously described (Okkenhaug et al., 2002). Female mice were used at 8–12 weeks old. All mice were bred at the University of Cambridge Central Biomedical Services under pathogen-free conditions and housed according to UK Home Office guidelines. Animal studies have been reviewed and approved by the UK Home Office.

Cell Line

A20 (BALB/c, H-2^d, B cell lymphoma) cells, a gift of Prof. K. Smith, were maintained in RPMI-1640 medium with stable glutamine and supplemented with penicillin/streptomycin (all PAA), 10% fetal bovine serum (Life Technologies), and 50 μM β-mercaptoethanol (Sigma).

Flow Cytometry

Conjugated monoclonal antibodies (mAbs) anti-mouse CD45 (30-F11), CD3ε (500A2 or 17A2), H-2K^b (AF6-88.5), H-2K^d (SF-1.1.1), CD62L (MEL-14), CD44 (IM7), CD25 (PC61), IFN-γ (XMG1.2), CD16/32-Fc blocking (93), granzyme B (GB11), FoxP3 (NRRF-30), P-Akt (Ser473) (D9E), and P-Akt (Thr308) (D25E6) and mAbs anti-human CD3 (UCHT1), CD45RA (HI100), CD45RO (UCHL1), CD25 (BC96), CCR7 (G043H7), CD8 (SK1), and human Fc blocking were purchased from BioLegend, eBioscience, BD Biosciences, or Cell Signaling. Granzyme B and FoxP3 were stained using the FoxP3 staining buffer set (eBioscience) according to the manufacturer's instructions. P-Akt was stained using the BD Phosflow Lyse/Fix buffer and Perm Buffer III following the BD Phosflow staining protocol. As negative controls for P-Akt, cells were pretreated for 30 min with the pan-PI3K inhibitor LY294002 (Cayman Chemical) or with the pan-Akt phosphorylation inhibitor MK-2206 (Selleckchem). DAPI (Life Technologies) or fixable viability dye eFluor 450 (eBioscience) was used to exclude dead cells. Samples were acquired on a LSR Fortessa (BD Biosciences) using FACS Diva (BD Biosciences) and analyzed using FlowJo (Tree Star).

GvHD and GvL

In GvHD experiments, lethally irradiated (2 × 4.5 Gy) BALB/c host mice were intravenously injected with 5 × 10^6 TCD BMCs from B6 mice alone (control) or along with purified T cells from WT, $p110\delta^{WT/D910A}$, or $p110\delta^{D910A}$ mice. Magnetic beads (CD3 Microbead kit, Pan T Cell Isolation kit II; Miltenyi Biotec) were used to deplete T cells in BMCs and to purify donor T cells from spleens (purity >90%). In experiments using sorted cells, naive T cells ($CD3^+$ $CD25^-CD62L^+CD44^{lo}$) and memory T cells ($CD3^+CD25^-CD44^{hi}$) were sorted using either a FACS Aria III or a FACS Aria Fusion (BD Biosciences) (purity >99%). In GvL experiments, 5 × 10^4 A20 cells were injected additionally. Mice were under antibiotic treatment (Baytril, Bayer) during the duration of the experiment. The clinical score was established at the time of sacrifice and based upon mouse weight loss (0–2), posture (0–2), activity (0–2), and fur texture (0–2), with the maximum clinical score being 8. Mice were culled before reaching 20% (Figure 1) or 25% (Figures 2 and 4) of weight loss depending on the protocol and according to UK Home Office-regulated procedures.

Allogeneic T Cells and BMDCs Coculture

Lipopolysaccharide (LPS)-matured BMDCs were generated as previously described (Lutz et al., 1999), then irradiated (21 Gy). Naive T cells and memory T cells were fluorescence-activated cell sorted, labeled with 5 μM of carboxyfluorescein succinimidyl ester (CFSE, Life Technologies), and cocultured with BMDCs (1:1 ratio) for 5 days in the presence of DMSO (Sigma-Aldrich), the p110δ-specific inhibitors IC87114 (UCB Cell Tech) and idelalisib (Selleckchem), or the pan-PI3K inhibitor LY294002 at the indicated doses. Then, T cell proliferation (CFSE dilution) was assessed by flow cytometry. Generation of alloreactive T cells was based on a previously described protocol (Sauer et al., 2004). In brief, naive and memory T cells were primed with irradiated (21 Gy) BALB/c BMDCs pulsed with A20 lysate, obtained by four freeze-thaw cycles, at a 20:1 ratio for 5 days. T cells were expanded 2 days further with plate-bound anti-CD3 antibody (clone 145-2C11) at 10 μg/ml, soluble anti-CD28 (37.51) at 2 μg/ml, rhIL-2 (Proleukin, Novartis) at 20 IU/ml, and rmIL-7 (Peprotech) at 4 ng/ml, then maintained in culture with cytokines only.

Human Cells and MLR Assay

PBMCs from healthy donors were isolated using a Histopaque-1077 gradient (Sigma). Total PBMCs or sorted naive and memory T cells from a donor were

labeled with 5 μM of CFSE, mixed with irradiated (30 Gy) PBMCs from another donor (1:1 ratio), cultured for 6 days in the presence of DMSO or PI3K inhibitors, and analyzed by flow cytometry.

Statistical Analysis

A two-tailed, unpaired Student's t test (Excel, Microsoft), Mantel-Cox test, or one-way ANOVA followed by Tukey's multiple comparisons test (GraphPad Prism 6) was used to statistically analyze the data, where $p < 0.05$ was considered significant. All data are expressed as means ± range.

SUPPLEMENTAL INFORMATION

Supplemental Information includes two figures and can be found with this article online at http://dx.doi.org/10.1016/j.celrep.2015.01.002.

AUTHOR CONTRIBUTIONS

J.-M.D. designed experiments and performed research; C.M.H. performed experiments; K.O. provided materials; C.M.H and K.O. helped with manuscript editing; and J.-M.D. and F.C. conceived the project, analyzed data, and wrote the manuscript.

ACKNOWLEDGMENTS

We thank the University of Cambridge Central Biomedical Services facility, the National Institute for Health Research Cambridge Biomedical Research Center Cell Phenotyping Hub and the members of the Colucci lab for helpful discussions. This work was supported by grants from Biotechnology and Biological Sciences Research Council, the Wellcome Trust (088621/Z/09/Z), and Leukaemia & Lymphoma Research (13010) to F.C.

Received: June 2, 2014
Revised: November 11, 2014
Accepted: December 24, 2014
Published: February 5, 2015

REFERENCES

Bian, G., Ding, X., Leigh, N.D., Tang, Y., Capitano, M.L., Qiu, J., McCarthy, P.L., Liu, H., and Cao, X. (2013). Granzyme B-mediated damage of CD8+ T cells impairs graft-versus-tumor effect. J. Immunol. *190*, 1341–1350.

Billottet, C., Grandage, V.L., Gale, R.E., Quattropani, A., Rommel, C., Vanhaesebroeck, B., and Khwaja, A. (2006). A selective inhibitor of the p110delta isoform of PI 3-kinase inhibits AML cell proliferation and survival and increases the cytotoxic effects of VP16. Oncogene *25*, 6648–6659.

Chan, W.K., Suwannasaen, D., Throm, R.E., Li, Y., Eldridge, P.W., Houston, J., Gray, J.T., Pui, C.H., and Leung, W. (2014). Chimeric antigen receptor-redirected CD45RA-negative T cells have potent antileukemia and pathogen memory response without graft-versus-host activity. Leukemia, Published online June 3, 2014. http://dx.doi.org/10.1038/leu.2014.174.

Chapman, P.B., Hauschild, A., Robert, C., Haanen, J.B., Ascierto, P., Larkin, J., Dummer, R., Garbe, C., Testori, A., Maio, M., et al.; BRIM-3 Study Group (2011). Improved survival with vemurafenib in melanoma with BRAF V600E mutation. N. Engl. J. Med. *364*, 2507–2516.

Chen, B.J., Deoliveira, D., Cui, X., Le, N.T., Son, J., Whitesides, J.F., and Chao, N.J. (2007). Inability of memory T cells to induce graft-versus-host disease is a result of an abortive alloresponse. Blood *109*, 3115–3123.

Distler, E., Bloetz, A., Albrecht, J., Asdufan, S., Hohberger, A., Frey, M., Schnürer, E., Thomas, S., Theobald, M., Hartwig, U.F., and Herr, W. (2011). Alloreactive and leukemia-reactive T cells are preferentially derived from naive precursors in healthy donors: implications for immunotherapy with memory T cells. Haematologica *96*, 1024–1032.

Dutt, S., Baker, J., Kohrt, H.E., Kambham, N., Sanyal, M., Negrin, R.S., and Strober, S. (2011). CD8+CD44(hi) but not CD4+CD44(hi) memory T cells mediate potent graft antilymphoma activity without GVHD. Blood *117*, 3230–3239.

Eagle, R.A., and Trowsdale, J. (2007). Promiscuity and the single receptor: NKG2D. Nat. Rev. Immunol. *7*, 737–744.

Edinger, M., Hoffmann, P., Ermann, J., Drago, K., Fathman, C.G., Strober, S., and Negrin, R.S. (2003). CD4+CD25+ regulatory T cells preserve graft-versus-tumor activity while inhibiting graft-versus-host disease after bone marrow transplantation. Nat. Med. *9*, 1144–1150.

Furman, R.R., Sharman, J.P., Coutre, S.E., Cheson, B.D., Pagel, J.M., Hillmen, P., Barrientos, J.C., Zelenetz, A.D., Kipps, T.J., Flinn, I., et al. (2014). Idelalisib and rituximab in relapsed chronic lymphocytic leukemia. N. Engl. J. Med. *370*, 997–1007.

Garçon, F., Patton, D.T., Emery, J.L., Hirsch, E., Rottapel, R., Sasaki, T., and Okkenhaug, K. (2008). CD28 provides T-cell costimulation and enhances PI3K activity at the immune synapse independently of its capacity to interact with the p85/p110 heterodimer. Blood *111*, 1464–1471.

Ghigo, A., Damilano, F., Braccini, L., and Hirsch, E. (2010). PI3K inhibition in inflammation: Toward tailored therapies for specific diseases. BioEssays *32*, 185–196.

Graubert, T.A., DiPersio, J.F., Russell, J.H., and Ley, T.J. (1997). Perforin/granzyme-dependent and independent mechanisms are both important for the development of graft-versus-host disease after murine bone marrow transplantation. J. Clin. Invest. *100*, 904–911.

Haarberg, K.M., Li, J., Heinrichs, J., Wang, D., Liu, C., Bronk, C.C., Kaosaard, K., Owyang, A.M., Holland, S., Masuda, E., et al. (2013). Pharmacologic inhibition of PKCα and PKCθ prevents GVHD while preserving GVL activity in mice. Blood *122*, 2500–2511.

Hamid, O., Robert, C., Daud, A., Hodi, F.S., Hwu, W.J., Kefford, R., Wolchok, J.D., Hersey, P., Joseph, R.W., Weber, J.S., et al. (2013). Safety and tumor responses with lambrolizumab (anti-PD-1) in melanoma. N. Engl. J. Med. *369*, 134–144.

Hoffmann, P., Ermann, J., Edinger, M., Fathman, C.G., and Strober, S. (2002). Donor-type CD4(+)CD25(+) regulatory T cells suppress lethal acute graft-versus-host disease after allogeneic bone marrow transplantation. J. Exp. Med. *196*, 389–399.

Houot, R., Kohrt, H., Goldstein, M.J., and Levy, R. (2011). Immunomodulating antibodies and drugs for the treatment of hematological malignancies. Cancer Metastasis Rev. *30*, 97–109.

Hubbard, V.M., Eng, J.M., Ramirez-Montagut, T., Tjoe, K.H., Muriglan, S.J., Kochman, A.A., Terwey, T.H., Willis, L.M., Schiro, R., Heller, G., et al. (2005). Absence of inducible costimulator on alloreactive T cells reduces graft versus host disease and induces Th2 deviation. Blood *106*, 3285–3292.

Janes, M.R., Limon, J.J., So, L., Chen, J., Lim, R.J., Chavez, M.A., Vu, C., Lilly, M.B., Mallya, S., Ong, S.T., et al. (2010). Effective and selective targeting of leukemia cells using a TORC1/2 kinase inhibitor. Nat. Med. *16*, 205–213.

Li, H.W., and Sykes, M. (2012). Emerging concepts in haematopoietic cell transplantation. Nat. Rev. Immunol. *12*, 403–416.

Li, J., Semple, K., Suh, W.K., Liu, C., Chen, F., Blazar, B.R., and Yu, X.Z. (2011). Roles of CD28, CTLA4, and inducible costimulator in acute graft-versus-host disease in mice. Biol. Blood Marrow Transplant. *17*, 962–969.

Li, J., Heinrichs, J., Leconte, J., Haarberg, K., Semple, K., Liu, C., Gigoux, M., Kornete, M., Piccirillo, C.A., Suh, W.K., and Yu, X.Z. (2013). Phosphatidylinositol 3-kinase-independent signaling pathways contribute to ICOS-mediated T cell costimulation in acute graft-versus-host disease in mice. J. Immunol. *191*, 200–207.

Lutz, M.B., Kukutsch, N., Ogilvie, A.L., Rössner, S., Koch, F., Romani, N., and Schuler, G. (1999). An advanced culture method for generating large quantities of highly pure dendritic cells from mouse bone marrow. J. Immunol. Methods *223*, 77–92.

McDaniel, J.M., Pinilla-Ibarz, J., and Epling-Burnette, P.K. (2012). Molecular action of lenalidomide in lymphocytes and hematologic malignancies. Adv. Hematol. *2012*, 513702.

Mellman, I., Coukos, G., and Dranoff, G. (2011). Cancer immunotherapy comes of age. Nature *480*, 480–489.

Ohata, J., Sakurai, J., Saito, K., Tani, K., Asano, S., and Azuma, M. (2002). Differential graft-versus-leukaemia effect by CD28 and CD40 co-stimulatory blockade after graft-versus-host disease prophylaxis. Clin. Exp. Immunol. *129*, 61–68.

Okkenhaug, K. (2013). Signaling by the phosphoinositide 3-kinase family in immune cells. Annu. Rev. Immunol. *31*, 675–704.

Okkenhaug, K., Bilancio, A., Farjot, G., Priddle, H., Sancho, S., Peskett, E., Pearce, W., Meek, S.E., Salpekar, A., Waterfield, M.D., et al. (2002). Impaired B and T cell antigen receptor signaling in p110delta PI 3-kinase mutant mice. Science *297*, 1031–1034.

Patton, D.T., Garçon, F., and Okkenhaug, K. (2007). The PI3K p110delta controls T-cell development, differentiation and regulation. Biochem. Soc. Trans. *35*, 167–171.

Putz, E.M., Prchal-Murphy, M., Simma, O.A., Forster, F., Koenig, X., Stockinger, H., Piekorz, R.P., Freissmuth, M., Müller, M., Sexl, V., and Zebedin-Brandl, E. (2012). PI3Kδ is essential for tumor clearance mediated by cytotoxic T lymphocytes. PLoS ONE *7*, e40852.

Robb, R.J., Lineburg, K.E., Kuns, R.D., Wilson, Y.A., Raffelt, N.C., Olver, S.D., Varelias, A., Alexander, K.A., Teal, B.E., Sparwasser, T., et al. (2012). Identification and expansion of highly suppressive CD8(+)FoxP3(+) regulatory T cells after experimental allogeneic bone marrow transplantation. Blood *119*, 5898–5908.

Ruggeri, L., Capanni, M., Urbani, E., Perruccio, K., Shlomchik, W.D., Tosti, A., Posati, S., Rogaia, D., Frassoni, F., Aversa, F., et al. (2002). Effectiveness of donor natural killer cell alloreactivity in mismatched hematopoietic transplants. Science *295*, 2097–2100.

Sauer, M.G., Ericson, M.E., Weigel, B.J., Herron, M.J., Panoskaltsis-Mortari, A., Kren, B.T., Levine, B.L., Serody, J.S., June, C.H., Taylor, P.A., and Blazar, B.R. (2004). A novel system for simultaneous in vivo tracking and biological assessment of leukemia cells and ex vivo generated leukemia-reactive cytotoxic T cells. Cancer Res. *64*, 3914–3921.

Sinclair, L.V., Finlay, D., Feijoo, C., Cornish, G.H., Gray, A., Ager, A., Okkenhaug, K., Hagenbeek, T.J., Spits, H., and Cantrell, D.A. (2008). Phosphatidylinositol-3-OH kinase and nutrient-sensing mTOR pathways control T lymphocyte trafficking. Nat. Immunol. *9*, 513–521.

Soond, D.R., Bjørgo, E., Moltu, K., Dale, V.Q., Patton, D.T., Torgersen, K.M., Galleway, F., Twomey, B., Clark, J., Gaston, J.S., et al. (2010). PI3K p110delta regulates T-cell cytokine production during primary and secondary immune responses in mice and humans. Blood *115*, 2203–2213.

Soond, D.R., Slack, E.C., Garden, O.A., Patton, D.T., and Okkenhaug, K. (2012). Does the PI3K pathway promote or antagonize regulatory T cell development and function? Front. Immunol. *3*, 244.

Stetson, D.B., Mohrs, M., Reinhardt, R.L., Baron, J.L., Wang, Z.E., Gapin, L., Kronenberg, M., and Locksley, R.M. (2003). Constitutive cytokine mRNAs mark natural killer (NK) and NK T cells poised for rapid effector function. J. Exp. Med. *198*, 1069–1076.

Sujobert, P., Bardet, V., Cornillet-Lefebvre, P., Hayflick, J.S., Prie, N., Verdier, F., Vanhaesebroeck, B., Muller, O., Pesce, F., Ifrah, N., et al. (2005). Essential role for the p110delta isoform in phosphoinositide 3-kinase activation and cell proliferation in acute myeloid leukemia. Blood *106*, 1063–1066.

Taylor, P.A., Panoskaltsis-Mortari, A., Freeman, G.J., Sharpe, A.H., Noelle, R.J., Rudensky, A.Y., Mak, T.W., Serody, J.S., and Blazar, B.R. (2005). Targeting of inducible costimulator (ICOS) expressed on alloreactive T cells down-regulates graft-versus-host disease (GVHD) and facilitates engraftment of allogeneic bone marrow (BM). Blood *105*, 3372–3380.

Teschner, D., Distler, E., Wehler, D., Frey, M., Marandiuc, D., Langeveld, K., Theobald, M., Thomas, S., and Herr, W. (2014). Depletion of naive T cells using clinical grade magnetic CD45RA beads: a new approach for GVHD prophylaxis. Bone Marrow Transplant. *49*, 138–144.

Touzot, F., Neven, B., Dal-Cortivo, L., Gabrion, A., Moshous, D., Cros, G., Chomton, M., Luby, J.M., Terniaux, B., Magalon, J., et al. (2014). CD45RA depletion in HLA-mismatched allogeneic hematopoietic stem cell transplantation for primary combined immunodeficiency: A preliminary study. J. Allergy Clin. Immunol. http://dx.doi.org/10.1016/j.jaci.2014.08.019

Valenzuela, J.O., Iclozan, C., Hossain, M.S., Prlic, M., Hopewell, E., Bronk, C.C., Wang, J., Celis, E., Engelman, R.W., Blazar, B.R., et al. (2009). PKCtheta is required for alloreactivity and GVHD but not for immune responses toward leukemia and infection in mice. J. Clin. Invest. *119*, 3774–3786.

Wolchok, J.D., Kluger, H., Callahan, M.K., Postow, M.A., Rizvi, N.A., Lesokhin, A.M., Segal, N.H., Ariyan, C.E., Gordon, R.A., Reed, K., et al. (2013). Nivolumab plus ipilimumab in advanced melanoma. N. Engl. J. Med. *369*, 122–133.

Yang, Y., Wang, H., Yu, H., Yeap, B.Y., Liang, T., Wang, G., Cheng, T., and Yang, Y.G. (2011). IFN-γ promotes graft-versus-leukemia effects without directly interacting with leukemia cells in mice after allogeneic hematopoietic cell transplantation. Blood *118*, 3721–3724.

Zhang, Y., Joe, G., Hexner, E., Zhu, J., and Emerson, S.G. (2005a). Alloreactive memory T cells are responsible for the persistence of graft-versus-host disease. J. Immunol. *174*, 3051–3058.

Zhang, Y., Joe, G., Hexner, E., Zhu, J., and Emerson, S.G. (2005b). Host-reactive CD8+ memory stem cells in graft-versus-host disease. Nat. Med. *11*, 1299–1305.

Cell Reports
Supplemental Information

Immunomodulation of Selective Naive T Cell Functions by p110δ Inactivation Improves the Outcome of Mismatched Cell Transplantation

Jean-Marc Doisne, Christian M. Hüber, Klaus Okkenhaug, and Francesco Colucci

Figure S1 – Related to Figure 3

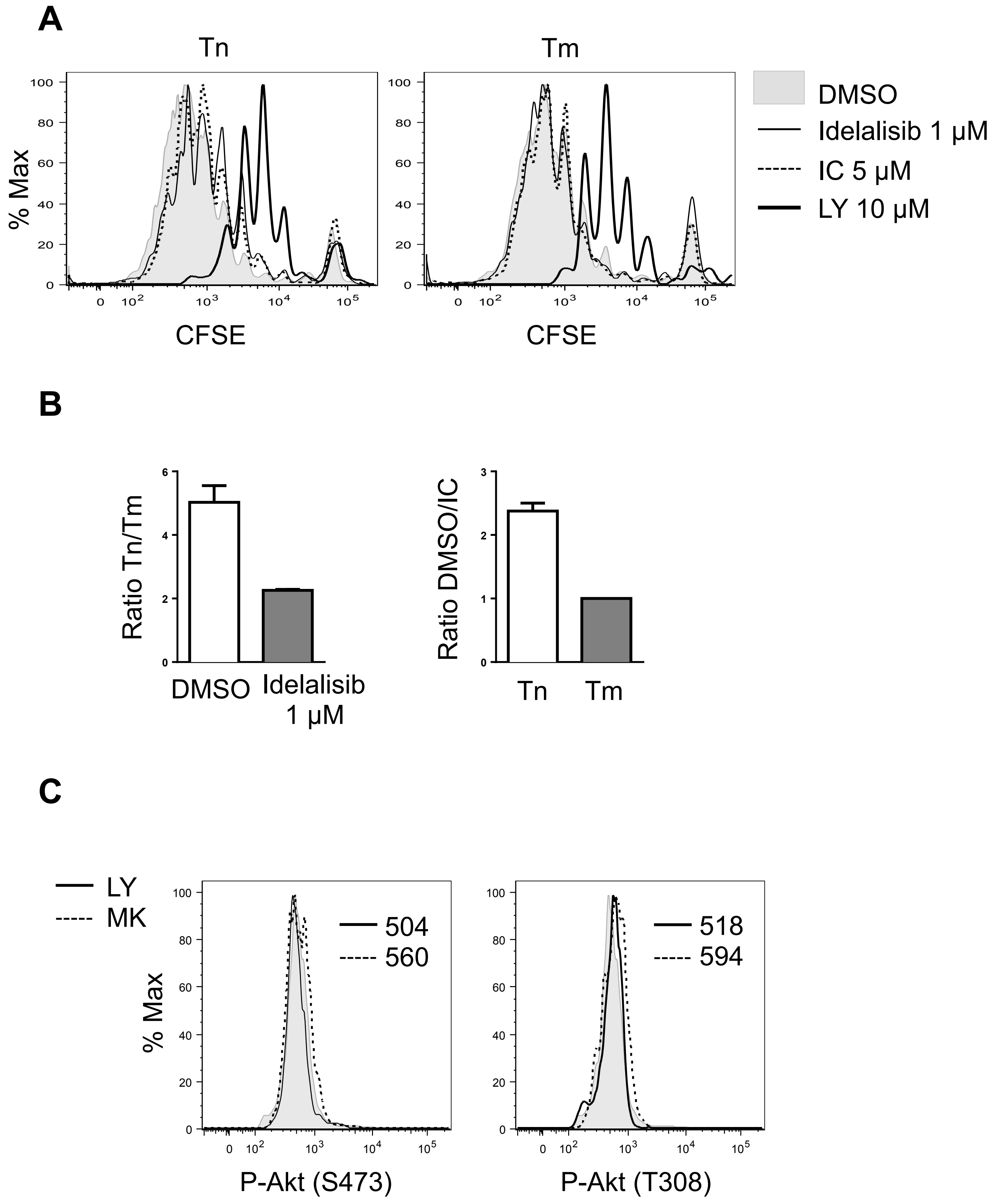

Figure S2 – Related to Figure 4

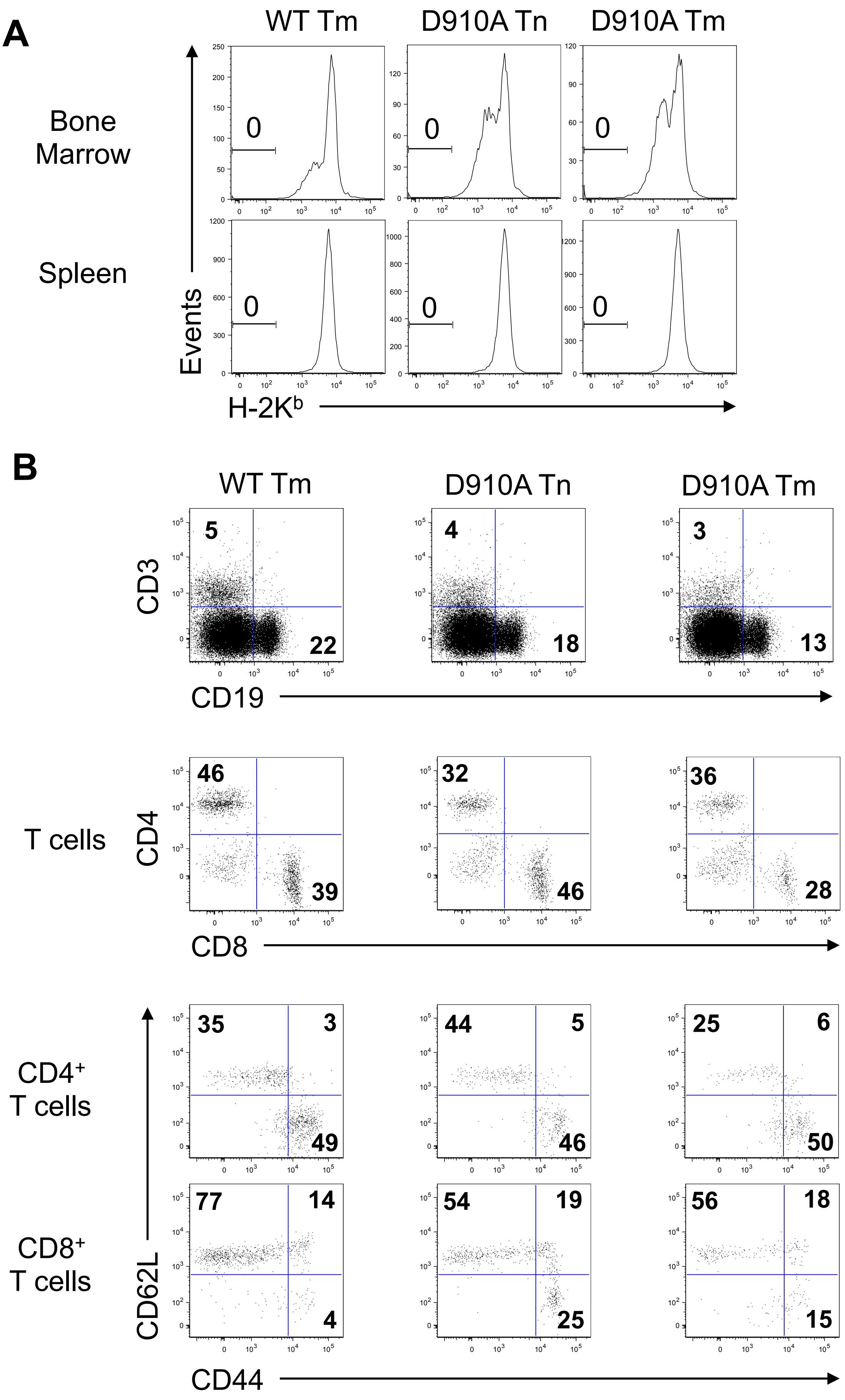

SUPPLEMENTAL LEGENDS

Figure S1 – Related to Figure 3. (A) Same experiment as in **Fig 3A** including treatments with DMSO (vehicle), Idelalisib (1 µM), IC87114 (5 µM) and LY294002 (10 µM). Results are representative of two independent experiments. **(B)** Alloreactive T cells were generated as in **Fig 3C** in the presence of DMSO or Idelalisib (1 µM). Results are presented as in **Fig 3C** and are representative of two independent experiments. **(C)** Alloreactive Tn generated in culture in the presence of DMSO were pre-treated with LY294002 (10 µM) or MK-2206 (5 µM) for 30 min then stained for P-Akt (Ser473) or P-Akt (Thr308). Results are representative of two independent experiments.

Figure S2 – Related to Figure 4. (A-B) Surviving mice from WT Tm group, D910A Tn group and D910A Tm group (depicted in Fig. 4) were analyzed by flow cytometry at day 91. **(A)** Histograms show representative examples of the absence of A20 cells (H-2K^{b-} CD19^{+}) in the BM and the spleen. **(B)** Dot plots show representative examples of leukocyte composition in the BM: T and B cells (upper panel), CD4^{+}/CD8^{+} T cells (middle panel) and their differentiation phenotype (bottom panel).
